# Barriers to hand hygiene practice among healthcare workers in health centres of Kirkos and Akaki Kality sub-cities, Addis Ababa, Ethiopia: a qualitative study

**DOI:** 10.1016/j.infpip.2025.100450

**Published:** 2025-02-28

**Authors:** Asmamaw Deguale Worku, Abayneh Melaku

**Affiliations:** a Department of Water and Health, Ethiopian Institute of Water Resources, Addis Ababa University, Addis Ababa, Ethiopia; b Department of Public Health Emergency Management, Addis Ababa Health Bureau, Addis Ababa, Ethiopia

**Keywords:** Barriers, Hand hygiene, Healthcare workers, Qualitative study, Addis Ababa, Ethiopia

## Abstract

**Background:**

Hand hygiene is an easy step to reduce healthcare-acquired infections, and improve patient safety. Major progress in facility accessibility was made during the coronavirus disease 2019 (COVID-19) pandemic, and this led to greater adherence to hand hygiene practices. However, handwashing practices have now returned to the pre-COVID-19 era. Most of the studies that have investigated hand hygiene adherence are quantitative. As such, this study aimed to explore barriers to hand hygiene practice in the health centres of Kirkos and Akaki Kality sub-cities in Addis Ababa, Ethiopia.

**Methods:**

Twenty-four healthcare professionals employed at the aforementioned health centres were interviewed using the key informant interview method. Data were analysed using a qualitative content analysis technique.

**Results:**

Based on the findings, there were three main categories of barriers to hand hygiene practice: barriers related to individuals (including three sub-categories: lack of knowledge and skill, improper attitude, and poor attention and negligence of healthcare workers); leadership barriers (including two sub-categories: lack of dedicated staff and low attention of leaders); and institutional barriers (including three sub-categories: inappropriate infrastructure and lack of resources, shortage of water, and high work load and staff turnover).

**Conclusion:**

There were several reasons why hand hygiene guidance was not followed. Hand hygiene barriers can be minimized by displaying colour-coded notice boards, making washing facilities easily accessible, monitoring the availability of soap, offering training, and providing accurate evidence about the need to enhance hand hygiene.

## Introduction

Hand hygiene is an easy way to reduce nosocomial infection, prevent the transmission of antibiotic-resistant bacteria, and improve patient safety [1]. Half of all nosocomial illnesses could be prevented with simple handwashing [2]. Healthcare-acquired infections increase hospital stays, have a significant financial impact on patients and the community [3], and can result in deaths. In developing nations, healthcare-associated infections (HCAIs) increase morbidity and mortality rates significantly, placing additional strain on already scarce resources [4].

Increased clinical expenses, use of medication, and hospital stays are linked to nosocomial infections. Hospital-acquired infections are preventable with the use of infection prevention and control (IPC) measures [5]. Healthcare professionals often come into contact with patients and their surroundings, which increases the risk of bacteria spreading via their hands.

Adherence to hand hygiene practices can reduce infections, hospital stays and mortality risk whilst improving patient health and safety. A meta-analysis suggested that handwashing with soap can reduce the risk of diarrhoeal disease by 23–48% [6,7], and reduce the risk of respiratory infections by 21–23% [7]. Although hand hygiene procedures are straightforward, many find them difficult to follow, and multiple studies have revealed that healthcare professionals adhere to and accept these practices poorly [8,9].

A study conducted in Indonesia to explore the determinants of handwashing with soap showed that a desire to smell nice, interpersonal influences, presence of handwashing places, and key handwashing moments (i.e. hands feeling dirty, including after eating and after cleaning child stools) were determinants of handwashing [10]. A study conducted in Northeast Ethiopia to assess hand hygiene compliance revealed that attending training on hand hygiene protocols, access to adequate soap and water, having alcohol for hand rubs, and having a handwash sink were factors associated with hand hygiene compliance [11]. Based on the findings in Northwest Ethiopia, knowledge about hand hygiene compliance, receiving training, presence of individual towels or tissue paper, presence of alcohol-based hand rubs, and presence of IPC committees were associated with hand hygiene practice [12]. A study conducted in East Ethiopia to assess factors of handwashing practice showed that having an educational status of grade 8 or more; living in an urban area; having role models, teachers and health professionals; availability of handwashing facilities; access to soap and water; and being a member of a water sanitation and hygiene (WASH) team were associated with good handwashing practice [13]. Research conducted in the municipality of Arba Minch revealed that handwashing practices were related to referent pressure, availability, and ease of access to soap and water [14].

Despite great improvement in hand hygiene accessibility during the coronavirus disease 2019 (COVID-19) pandemic, which led to greater compliance with hand hygiene practices, handwashing practices have now returned to the pre-COVID-19 era. Paradoxically, the majority of research that has looked at hand hygiene compliance has been quantitative. The behaviour related to handwashing is complex and challenging to clarify, alter or change. As such, it is necessary to apply qualitative research methods to investigate individuals' beliefs, attitudes, experiences, behaviours and intentions. Therefore, the aim of this study was to explore barriers to hand hygiene practice among healthcare workers in the health centres of Kirkos and Akaki Kality sub-cities in Addis Ababa, Ethiopia.

## Methods

### Study design

This study had a qualitative study design and applied conventional content analysis. A qualitative study is a crucial instrument for studying behaviours, emotions, feelings and human experience, which cannot be addressed through a quantitative study. The methodical coding and categorization process known as ‘content analysis’ is employed to comprehend, evaluate and frame the fundamental ideas of qualitative records.

### Sample size and setting

Healthcare providers working at Kasanchis, Efoyita, Gelan, Saris, and Kality health centres were included in the study using purposive sampling. The participants were entered into the study from emergency and injection service points, with a total of 24 participants. Key informant interviews were performed, in which the participants were interviewed using semi-structured probing questions, and were willing and able to share their experiences. All methods were performed in accordance with the relevant guidelines and regulations. The inclusion criteria required healthcare workers to have at least 1 year of experience in a health centre. The study excluded participants with a history of mental illness and those with a critical illness. The sample size of a qualitative study depends on data saturation, which determines whether sufficient data are present to form a comprehensive understanding [15,16]. In this study, data saturation was reached after 21 participants had been interviewed; however, three additional interviews were conducted to confirm data saturation [17].

### Data collection process

Semi-structured interviews using open-ended questions were used to gather data from 1^st^ April to 30^th^ June 2024. Each interviewed participant was interviewed once. Interviews took place in the morning in a single room. To establish a relationship and familiarize the researcher with the participant, several preparatory questions were asked at the beginning of the interview. An interview guide was utilized to elicit further information from the participants during the interview procedure. The interviews then focused on the study's objective. The duration of the interviews ranged from 43 to 65 min, and the researcher encouraged healthcare professionals to engage in dialogue and share their experiences. Examples of questions include: Which amenities can you use to wash your hands in your room? According to you, for which five periods should one practice good hand hygiene? Would you please share your experience with hand hygiene difficulties encountered when providing care?

### Data analysis

Qualitative content analysis was performed to analyse the data following the five-step method of Hsieh and Shannon, and Lundman and Graneheim [[18], [19], [20]]. This technique is used to investigate typescripts, and finds codes and categories using a methodical classification process. Content analysis is beyond the mere abstraction of real content from written information. It helps to find hidden categories and patterns in the text produced by participants. In the first step, every interview was transcribed promptly. To fully immerse the researchers in the data and gain a general understanding of the material, the interview texts were reviewed multiple times. To manage the data, the text from each interview was entered into MAXQDA. Interview texts were studied in their entirety in the second step, in order to identify the meaning units pertinent to the study's objective. The meaning units were consolidated and given pertinent codes in the third step. In the fourth step, categories (and sub-categories) were created by sorting and organizing the codes into clusters based on the relationships between the codes. In the fifth step, the data's latent content was extracted. At the time of data collection and analysis, the researcher recorded any sparks related to the data, and used them for subsequent interviews. ADW analysed the interviews, and contributed to composition, review and correction of the final written report [19]. The authors reviewed and approved every extracted category and sub-category, and some of the analysis parts of content analysis (as indicated in Table I).

**Table I:** Qualitative content analysis of some parts of the analysis

Category	Sub-category	Code	Meaning unit
Individual barriers	Lack of knowledge and skill	Lack of awareness Unable to perform handwashing Not able to perform techniques of handwashing Unable to recall the proper time to wash their hands Low-practising health professionals at school Shortage of training Wear gloves for a long time	The healthcare workers were not aware of handwashing practices, and they had a knowledge gap about how much handwashing protected them from infectious diseases. The healthcare professionals were unable to perform handwashing scientifically; they simply washed their hands ordinarily; they did not utilize handwashing techniques; and they did not know when to wash. The healthcare professionals face low practice at school as well as a lack of on-the-job training and orientation in the health centres. Healthcare professionals presume that when gloves are worn, there is no need to wash hands. Wearing gloves for extended periods without remembering to wash hands.

Guba and Lincoln's criteria, including credibility, dependability, transferability and conformability, were used to ensure trustworthiness [21,22]. Numerous methods were used to ensure the data's credibility, including the researchers' prolonged presence at the site and in the field. Background information regarding the researcher, data collection techniques, procedure, data management, transcripts, translation, data analysis, procedure and research findings was evaluated as peer-reviewed processes. In addition, participants were required to take part in a member check, in which they read a brief report that contained the data that had been processed and interpreted. This action was undertaken to ensure that the outcomes reflected their circumstances and experiences accurately. Transferability of the findings was guaranteed by providing a detailed account of every stage of the data collection procedure, context, analysis, and direct quotes from participants. Additionally, the maximum sampling variability was considered, as well as saturation of the idea. Saturation was established when three concurrent interviews were conducted, and no new codes were identified [17,23].

### Ethical approval and consent to participate

Ethical approval was obtained from the Addis Ababa Health Bureau ethical review committee (AAT/14779/227). Informed agreement from the participants and data confidentiality throughout the analysis were among the ethical considerations of this investigation. After selection of the participants, the study objectives were explained to the participants, and informed and written consent was obtained for audio and text recording at the beginning of the interview. Participants were given assurances regarding the confidentiality of their data, and their ability to enter and exit the study. Interviews took place in person at the health centres at a designated time and location, with each participant identified by a pseudonym.

## Results

### Sample characteristics

This study involved 24 healthcare workers working at the health centres, and the interviews lasted for 43–65 min. The healthcare workers included general practitioners (GPs) (*N*=4), nurses (*N*=8), health officers (*N*=7) and environmental health (*N*=5). Fourteen men and 10 women participated in the study, and their length of work experience ranged from 3 to 17 years (Table II).

**Table II:** Characteristics of participants

Subject no.	Pseudonym	Gender (M, F)	Experience (years)	Profession
1	Pt 1	M	14	GP
2	Pt 2	F	8	Environmental health
3	Pt 3	M	3.4	Nurse
4	Pt 4	M	9	Health officer
5	Pt 5	F	3	Nurse
6	Pt 6	M	7	GP
7	Pt 7	M	13	Nurse
8	Pt 8	F	6	Nurse
9	Pt 9	M	15	Environmental health
10	Pt 10	F	5	Health officer
11	Pt 11	M	9	Health officer
12	Pt 12	M	14	Environmental health
13	Pt 13	F	8	Health officer
14	Pt 14	M	3.4	GP
15	Pt 15	M	9	Health officer
16	Pt 16	F	3	Nurse
17	Pt 17	M	7	Health officer
18	Pt 18	F	13	Environmental health
19	Pt 19	F	6	Environmental health
20	Pt 20	M	17	GP
21	Pt 21	F	5	Nurse
22	Pt 22	M	9	Nurse
23	Pt 23	F	3	Health officer
24	Pt 24	M	17	Nurse

Pt, participant; F, female; GP, general practitioner; M, male.

### Findings

There were three broad classifications and eight sub-categories: individual barriers (sub-categories: lack of knowledge and skills, poor attitude, low attention and negligence), leadership barriers (sub-categories: lack of dedicated staff, low attention of leaders), and institutional barriers (sub-categories: inappropriate infrastructure and shortage of resources, shortage of water, high work load and staff turnover). A summary of the categories and sub-categories is presented in Table III.

**Table III:** Barriers to hand hygiene practice in critical care point areas of health centres in 2024

Main category	Category	Sub-category
Barriers to hand hygiene practice	Individual barriers	Lack of knowledge and skills
		Poor attitude
		Low attention and negligence
	Leadership barriers	Lack of dedicated staff
		Low attention of leaders
	Institutional barriers	Inappropriate infrastructure and resources
		Shortage of water
		High work load and staff turnover

### Barriers to hand hygiene practice

Three categories were identified from analysis of the interviews: individual barriers, leadership barriers and institutional barriers. These are discussed in more detail below.

#### Individual barriers

This category comprised three subcategories, each of which is detailed below.

##### Lack of knowledge and skill

Half of the participants working at the health centres were unaware of the negative health impacts of poor hand hygiene practices, including antibiotic resistance, long duration of stay, and economic loss. The lack of awareness of staff, especially newcomers, contributed to non-adherence to handwashing practices. Participants assumed that hand hygiene was unnecessary as there was no visible hand contamination during caregiving or because gloves were used, which resulted in decreased hand hygiene practice:

‘I don't want to wash my hands after every activity and patient touch because I'm not sure how much it will protect me’ (Pt 3).

‘I assume my hands are clean since I handled cold patients and never wash them while I'm in a debilitated state’ (Pt 14).

More than half of the participants did not know how or when to perform hand hygiene in the healthcare setting, and simply washed their hands as usual if they were visibly dirty:

‘I was offended for various reasons and didn't care about it, so I didn't follow the proper handwashing techniques’ (Pt 21).

##### Poor attitude

The majority of participants' experiences demonstrated that the unfavourable attitudes and beliefs of healthcare professionals regarding hand hygiene practices had a significant impact on non-adherence due to pessimism, low motivation and poor perception. As a result, a negative attitude regarding handwashing could have a significant impact on other staff members at the health centres, which could result in a decline in hand hygiene compliance. Although hand hygiene showed significant improvement during the COVID-19 pandemic, it is still dependent on individual perceptions, beliefs and behaviours:

‘It is easy to wash one's hands, but most health staff do not believe that handwashing is necessary and their numerous instances of negligence’ (Pt 1).

‘Although washing my hands is a simple process, I don't make it a habit. Humans are more likely to engage in bad habits and behaviours than in positive ones. The health professional also had deprived boldness to washing practice. Handwashing is not difficult to do, yet it is often overlooked due to ignorance of its significance’ (Pt 5).

‘The medical experts washed their hands as continually as they assumed they should own, rather than as often as was essential’ (Pt 11).

##### Low attention and negligence of health professional

Most participants mentioned that poor kindness and carelessness of healthcare workers were barriers to hand hygiene practice in the area. This practice was influenced by many factors, including poor attention, ignorance and carelessness of healthcare workers:

‘Handwashing is neglected in the weekend time because of emergencies and cold cases come together and manage them without washing hands. In my opinion, the case is not as such infectious will continue the work without handwashing’ (Pt 17).

‘It is not a knowledge gap, but rather Meselachet or medical staff negligence that prevents people from washing their hands’ (Pt 13).

‘The health staff did not need to wash their hands due to carelessness, absence of devotion, and non-existence of culpability’ (Pt 7).

#### Leadership barriers

This category focused on leadership and administrative styles of the institution that affect hand hygiene. In most cases, the respondents addressed the lack of dedicated staff to control the practice, and the low attention of leaders. Leaders can improve washing practices at health centres via the deployment of sufficient staff and due attention.

##### Lack of dedicated staff

Some participants reported that the lack of dedicated IPC-WASH staff exerts negative pressure on handwashing practice:

‘Due to a shortage of designated health professionals, no one provided us with guidance, coaching, or monitoring to determine whether we were practising handwashing correctly or not. Additionally, no supervision was carried out during handwashing’ (Pt 9).

##### Low attention of leaders

Most respondents recognized that low attention of leaders is a barrier to hand cleanliness. Low engagement of higher officials and leaders in the design and construction of health centres, availability of handwashing facilities, and low commitment to the accessibility of essential materials for handwashing lead to non-adherence to handwashing practice:

‘The building was not initially constructed for health centre rather for other purpose, and cannot install water pipeline and handwashing facility because of low attention and commitment of leaders’ (Pt 10).

‘In my opinion, handwashing is not as such practiced, because of low commitment and low attention from management and leaders’ (Pt 11).

‘A lack of commitment and responsibility has resulted in poor leadership involvement, poor direction at construction, and poor messaging among the construction bureau and the health sector’ (Pt 16).

#### Institutional barriers

Institutional barriers were those arising from the health centre arrangement, infrastructure or environment. Several participants addressed inappropriate infrastructure of the health centres and lack of resources, shortage of water, and high work load and staff turnover as obstacles to handwashing.

##### Inappropriate infrastructure and lack of resources

Most of the participants felt that inappropriate infrastructure and shortage of resources were the most substantial barriers to hand hygiene practice in this setting. If the healthcare facility experienced shortages of the necessary resources to wash hands (e.g. soap, alcohol-based hand rub and 0.05 chlorine solution), the participants believed that this contributed to poor hand hygiene compliance. In addition, the distance of the handwashing facilities from the service delivery point was a significant barrier to hand hygiene practice. Theft of dry soap is a factor that leads to non-compliance with the handwashing practice of medical staff working at the health centre:

‘First, the health centre is designed for other purposes, like a hotel, so it makes it difficult to access handwashing facilities. Second, the construction design of the health centre is not suitable for the installation of handwashing facilities’ (Pt 12).

‘Performing cleanliness of the hands is simple to follow; it confronts difficulties when the facility is not there’ (Pt 5).

‘Because it is not incorporated during building, poor infrastructure makes it difficult to install water pipelines’ (Pt 4).

##### Shortage of water

Most participants described shortage of water at the health centres as a barrier to hand hygiene practice. Lack of water at service delivery points leads to non-adherence to handwashing practices:

‘The washing practice is very poor because of shortage of water in the health centre, only 2 days in the week water comes Tuesday and Thursday, so it makes difficult to wash our hands’ (Pt 3).

##### High work load and staff turnover

High work load and staff turnover are crucial problems in practising hand hygiene, due to stress, a dearth of involvement, congestion, forgetting and rushing to finish the tasks assigned. Participants stated that, when providing care for multiple patients, often caring for two cases simultaneously, the practice is inevitably forgotten. Participants also noted that the impossibility of keeping away from critically ill patients was an additional obstacle to handwashing practice. In addition, exhaustion as a result of the high work load and staff turnover prohibited them from practising appropriate handwashing:

‘The medical professionals neglected to wash their hands during a client and customer overflow due to the urgency of the situation and the need to act quickly’ (Pt 2).

‘Overload at work. Handwashing becomes challenging when 45 cases are seen by one medical expert each day’ (Pt 1).

‘Due to the high volume of patients and intricate nature of the cases, the medical staff was unable to wash their hands in between cases because the water was located in a remote location, causing service interruptions’ (Pt 24).

## Discussion

This study explored the barriers to hand hygiene in Addis Ababa health centres through key informant interviews. Twenty-four semi-structured interviews resulted in identification of three categories and eight sub-categories: (1) individual barriers (sub-categories: knowledge and skills, poor attitude, low attention and negligence), (2) leadership barriers (sub-categories: lack of dedicated staff, low attention of leaders) and (3) institutional barriers (sub-categories: staff turnover and high work load, inadequate resources, inadequate infrastructure, lack of water). Subsumed within these categories are unique findings that have not been reported previously. These include lack of dedicated staff to monitor handwashing practices by the so-called ‘IPC-WASH’ officer, the health centre being constructed initially for another purpose, and lack of consultation of the design of the building for its premises according to the health facility requirements.

Many inadequate handwashing habits were due to the ignorance of health practitioners. Many of the medical staff at the health centres were unaware of the detrimental effects of poor hand hygiene practices on health, such as increased mortality, prolonged hospital stays and financial loss. Furthermore, impediments to handwashing practices included the absence of evident hand contamination and the use of alternate gloves. Concurrent findings from other research supported the finding from the present study that a lack of awareness acted as a deterrent to handwashing and contributed to non-compliance [[24], [25], [26]]:

‘I don't know how much washing my hands will protect me’.

‘I always don't wash my hands in the weekend time (Saturday and Sunday) because I managed cold cases as it is clean’.

A small percentage of participants were also unaware of the correct timing and technique for handwashing in a medical setting. This suggests that health personnel lack the necessary knowledge and proficiency to wash their hands properly. The knowledge and abilities of health professionals about hand cleanliness can be greatly enhanced by providing them with training on procedures and strategies [27,28].

Due to the incorrect views of healthcare professionals regarding handwashing methods, there was a decline in adherence to hand hygiene compliance. A correlation has been shown between favourable attitudes among healthcare personnel and an increase in handwashing behaviours [3,29]. Although handwashing became the norm during the height of the COVID-19 pandemic and the local epidemic, it later reverted to its pre-pandemic state. Due to their negative attitudes, actions and beliefs toward handwashing, healthcare workers are now practising handwashing less frequently than during the pandemic. Research revealed that healthcare professionals with positive attitudes adhered to handwashing protocols [30,31]. Thus, the development of training programmes is essential to improve the positive outlook of healthcare providers regarding handwashing habits, and control health attitudes to improve hand hygiene [32].

Studies have indicated a connection between non-compliance with handwashing practices and inattention and negligence [33]. One participant expressed the opinion that healthcare workers' inattention and irresponsibility, rather than lack of understanding, were to blame for the lack of handwashing. This suggests that poor adherence to hand hygiene practices and a route for the spread of infectious diseases were caused by healthcare workers' disregard for, and carelessness about, washing their hands in a medical setting. Thus, a focus on improving people's inattention to and carelessness about hand cleanliness would be given [34].

It was felt that facility administrators should have more influence over removing obstacles, and that they should be accountable for maintaining good handwashing procedures. One major barrier to handwashing practices at the healthcare centres was the lack of dedicated staff to oversee the practice [35]. Additional research corroborates the conclusion that management and leadership influence handwashing behaviour [25,36]. The health professionals were working without supervision or control, and the IPC-WASH experts were not following the handwashing protocol that the healthcare workers followed in the health facility. Thus, medical staff continue without oversight or assistance. Supervisors and managers in the health sector should play a crucial role in assigning IPC-WASH experts to health centres to enhance IPC practices, such as the hand hygiene practices of healthcare workers [25].

The main obstacle to hand hygiene practice is the lack of engagement shown by leaders and administrators of health centres. The management are not concerned with how handwashing techniques used at the centres influence other health professionals who choose to follow them, largely because many of the centre's employees look to them as role models. According to previous studies, the main challenge in hand hygiene compliance is supervisors' contempt for handwashing [37,38]. The lack of involvement of higher officials and leaders in the health centre's design and construction, the absence of handwashing stations, and the lack of dedication to ensuring that the required materials are easily accessible were all significant factors in non-adherence to practising hand hygiene. Thus, it would seem that management and leadership need to encourage handwashing [37,39].

A lack of resources and inadequate infrastructure were cited as obstacles to the practice of hand hygiene, despite the fact that the workforce was adequately trained and prepared to follow hand hygiene protocols. Sadly, they were unable to adhere to the advised hand hygiene practice due to being too far from the handwashing facility, the poor accessibility of sinks, and the lack of necessary supplies [40]. Similar to the current study, several previous investigations have identified inadequate infrastructure and a lack of resources as impediments to handwashing practice [32,[41], [42], [43], [44]]. To eliminate obstacles and boost adherence to hand hygiene practices, suitable cleaning supplies such as soap, alcohol-based hand rub, chlorine solution and handwashing sinks were also taken into consideration [45]. It was felt that the primary causes of hand hygiene non-compliance were the absence of handwashing facilities, soap, alcohol-based hand rub and chlorine solution. Thus, it is important to give this proper attention to encourage hand cleaning.

The absence of water in the study institution is a major barrier to healthcare workers performing hand hygiene. According to previous research, a lack of water can also be a barrier to practising hand hygiene elsewhere [44]. The respondents informed the authors during the interviews that water only comes to their health centres on Tuesdays and Thursdays, making handwashing extremely difficult. Healthcare professionals disregarded hand hygiene protocols as being a result of this issue. In order to encourage proper hand hygiene practices, a continuous water supply should be provided as a priority [46].

One of the main obstacles to handwashing in emergency rooms is high work load and staff turnover. Participants in the current study stated that high work load and staff turnover made it difficult or impossible to wash their hands. Exhaustion and a high patient load were the primary causes of non-compliance with hand hygiene. This corroborates findings from other studies [31,47,48]. Further research has shown that healthcare workers believed they had insufficient time to wash their hands during crises, and were less concerned about hand hygiene procedures [31,35,[49], [50], [51], [52]]. As a result, despite knowing the proper handwashing technique, the high work load and staff turnover will prevent healthcare workers from practising hand hygiene. Consequently, it is imperative to develop the institutional ability to manage emergencies, modify high work loads, improve staff retention by applying different incentives and motivation, and promote hand cleanliness [31,49,53,54].

### Strengths and limitations of the study

The principal investigator participated directly in the data collection procedure, enhancing understanding of the participants' ideas, feelings and thoughts; this is important for better output. The study's limitations include its reliance on a single data collection technique, and the potential for researcher bias due to purposeful sampling, which mainly relies on the investigator's discretion when selecting study subjects.

## Conclusion

Non-adherence to hand hygiene practices can be caused by various issues, such as inadequate knowledge and skill, inappropriate behaviour and attitude, inattentiveness, lack of dedicated staff, insufficient attention from leaders, inadequate infrastructure, scarcity of resources, shortage of water, high work load and staff turnover. Barriers to handwashing can be reduced by displaying posters, providing easy access to a washing area, monitoring the supply of materials, improving staff retention using different means, and providing training and accurate evidence on the usefulness of improving handwashing.

## Author contributions

ADW designed the study, prepared the guide tool, conducted and analysed the interviews, wrote the main manuscript, performed reviews, and corrected the written version. AM designed and reviewed the study, transcribed the interview, and prepared the draft manuscript. Both authors read and approved the final version of the manuscript.

## Funding sources

None.

## Availability of data and materials

The datasets used and analysed in this study are available from the corresponding author on reasonable request.

## Conflict of interest statement

None declared.
