# Supplementary material for: Barriers to hand hygiene practice among healthcare workers in health centres of Kirkos and Akaki Kality sub-cities, Addis Ababa, Ethiopia: a qualitative study
Source: Infect Prev Pract. 2025 Feb 28;7(2):100450. doi: 10.1016/j.infpip.2025.100450 (PMC11952847; doi:10.1016/j.infpip.2025.100450)
Supplement: Multimedia component 1 [file mmc1.docx]

Guide tool with probing questions for qualitative study of Barriers to hand hygiene practice among health care providers in AA: as follow

My name is Asmamaw Deguale and a researcher in Addis Ababa Health Bureau. I am conducting a qualitative study on hand hygiene practice in Addis Ababa, Ethiopia. I know all you have a busy schedule and programs, but this study is important to understand the hand hygiene practice among health professionals in Addis Ababa health facilities and to prevent transmission of communicable disease to HCW, the patients and visitors (community) as well, and to take an effective measure. The health professionals who are working in emergency room/injection room/ are an eligible to this study. Informed and written consent were taken to assure willingness for audio and text recording. The information about study objective, the right to participate and withdraw any time from the study would be provided.

1. What facilities are provided in your premise or emergency room for hand hygiene practices? Make a list of all the hand sanitation materials that are available to you.

2. Can you define your understanding of the significance for hand hygiene?

3. What are the five instants that you believe that washing your hands/hand hygiene should be done? When do you wash your hands/hand hygiene?

4. Can you describe how to perform hand hygiene?

5. How would you explain the need of hand hygiene to patients, hospitals, coworkers, and yourself personally?

6. Do you find it easy or difficult to practice hand hygiene? What makes you believe that?

7. Could you elaborate on any obstacles or barriers you've previously encountered when you perform hands hygiene?

8. In spite of the obstacles, do you think you can continue to perform hand hygiene more often? If so, what is your plan? If not, why do you believe so?

9. What factors influences your decision to perform hand hygiene? (Timing, nature of treatment, patient type?)

10. Do you usually remember to perform hand hygiene, or do you tend to forget? When is this most likely to occur?

11. What material or resource limitations (such as sink, water, alcohol gel, and time) affect or interfere with your practice of hand hygiene?

12. Can you list any conflicting duties that can affect hands hygiene?

13. Are there any interventions in place at work to make sure to practice hand hygiene?

14. Could you please share your experience with obstacles to hand hygiene when providing care?

15. Do you have any additional information or insights regarding the practice of hand hygiene?

16. What suggestion or solution is there for the issue at hand?

17. After you have summarized all the important topics covered in the conversation, ask them if they have any further questions. Lastly, express gratitude for their time.

Thank you for your participation!!!
